# Incidental detection of pancreatic cancer by F-18-fluorodeoxyglucose positron emission tomography/computed tomography in a patient with hereditary breast and ovarian cancer syndrome

**DOI:** 10.1007/s13691-024-00705-2

**Published:** 2024-07-26

**Authors:** Naotaka Uchida, Miho Takehita, Takako Suda

**Affiliations:** 1 Department of Breast and Endocrine Surgery, Matsue City Hospital, 32-1 Noshira-tyou, Matsue, Shimane 690-8509 Japan; 2 Department of Medical Fenetics, Matsue City Hospital, 32-1 Noshira-tyou, Matsue, Shimane 690-8509 Japan

**Keywords:** Hereditary breast and ovarian cancer syndrome, Pancreatic cancer, Breast cancer, F-18-fluorodeoxyglucose positron emission tomography/computed tomography

## Abstract

Patients with hereditary breast and ovarian cancer syndrome (HBOC) are associated with an increased risk of developing pancreatic cancer (PC) than the general population. There is no consensus about the clinical value of F-18-fluorodeoxyglucose (FDG)-positron emission tomography/computed tomography (PET/CT) in patients with HBOC. We report a patient with HBOC in whom PC was detected incidentally by PET/CT. A 48-year-old woman complaining of a right breast mass sought evaluation at our hospital. Her older brother died of PC at 49 years of age. Histologic analysis of the breast mass revealed breast cancer (BC). FDG-PET/CT showed unanticipated FDG accumulation in the pancreas. Magnetic resonance cholangiopancreatography (MRCP) revealed a mass in the pancreas approximately 25mm in size. Endoscopic ultrasound guided-fine needle aspiration biopsy (EUS-FNA) demonstrated PC. Genetic testing showed a *BRCA2* pathologic variant [NM_000059.4(BRCA2): c.9076C > T (p.Gln3026Ter)]. She was referred to a university hospital and underwent surgery after neoadjuvant chemotherapy for PC. It is difficult to detect operable PC in most patients. The diagnostic utility of PET/CT for PC in high-risk patients, such as those with HBOC, is undetermined. Our case has demonstrated the clinical value of PET/CT in detecting incidental PC in HBOC patients.

## Introduction

Patients with *BRCA* pathologic variants, such as hereditary breast and ovarian cancer syndrome (HBOC), are associated with an increased risk of developing pancreatic cancer (PC) as well as breast cancer (BC) compared with the general population. In Japan, the cumulative risk of BC to 85 years of age is 72.5%. The odds ratio (OR) for BC in patients with *BRCA1* and *BRCA2* mutations is 16.1 and 10.9, respectively [1]. The cumulative risk of PC to 85 years of age is 16.0%. The OR for PC in patients with *BRCA1* and *BRCA2* mutations is 12.6 and 10.7, respectively [1].

The 2024 National comprehensive cancer network guideline (version 2) has described PC screening using annual contrast-enhanced magnetic resonance imaging (MRI)/magnetic resonance cholangiopancreatography (MRCP) and/or endoscopic ultrasound (EUS) [2]. However, there is no consensus of opinions regarding the significance of F-18-fluorodeoxyglucose (FDG)-positron emission tomography/computed tomography (PET/CT) in patients with HBOC.

Therefore, we report a patient with HBOC in whom PC was detected incidentally by PET/CT.

## Case presentation

**Fig. 1:**
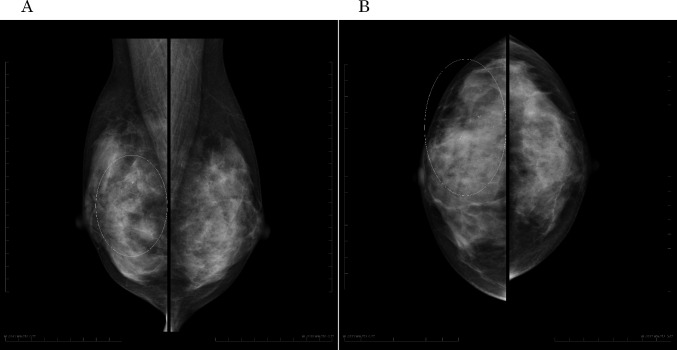
Mammography. Distortion and calcifications are circled. A: MLO view, B: CC view

**Fig. 2:**
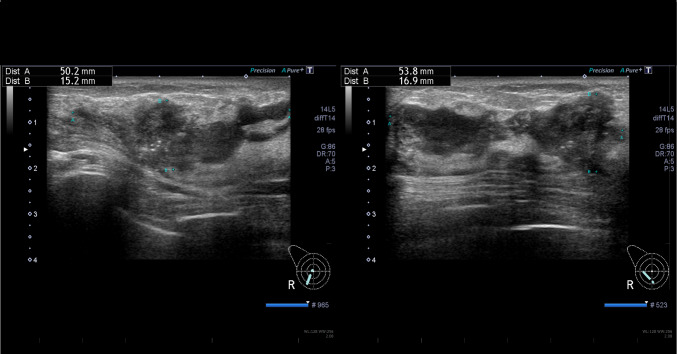
Ultrasonography. Tumor with ill-defined, irregular shape and low echogenicity was detected

**Fig. 3:**
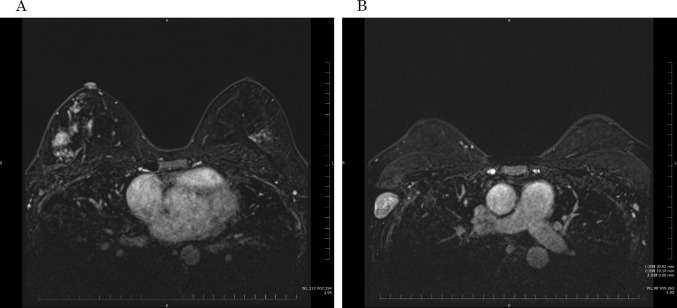
Breast MRI. Right breast nodules (**A**) and enlarged axillary lymph nodes (**B**) were noted

**Fig. 4:**
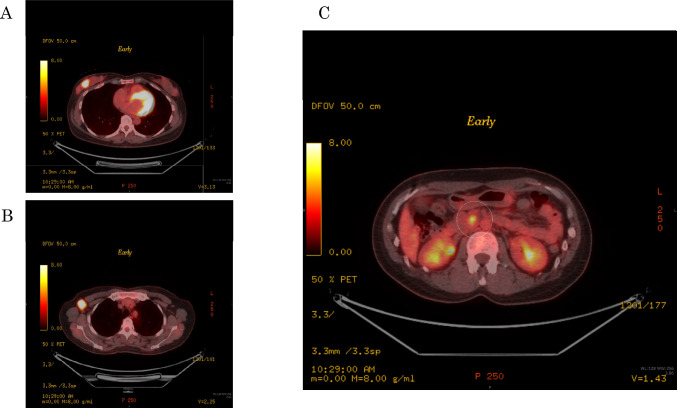
FDG-PET/CT. Accumulation is noted in the right breast (**A**), axillary lymph nodes (**B**) and pancreas head (C, circle)

**Fig. 5:**
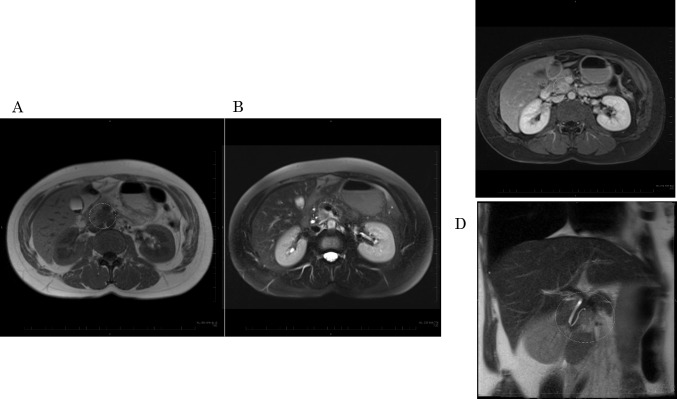
MRI. **A**: T1WI, **B**: T2WI, **C**: enhanced MRI, **D**: MRCP. **C**: Low-intensity in the early phase. High periphery intensity and iso-central intensity in the late phase. **D**: Mass is adjacent to the pancreatic duct but there is no stenosis

**Fig. 6:**
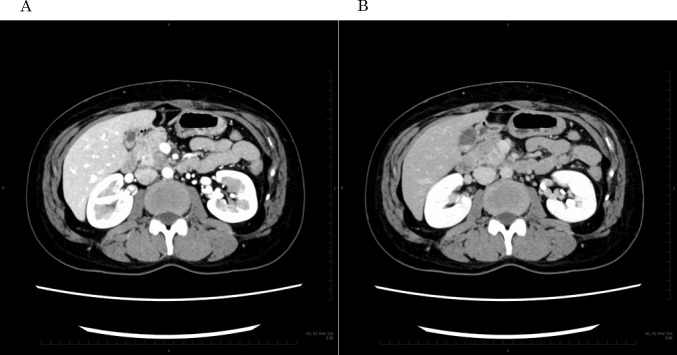
Contrast enhanced CT. **A**: early phase, **B**: late phase. Mass is gradually enhanced (circle)

A 48-year-old nulligravida sought evaluation at our hospital for a self-detected right breast mass. The medical history was benign. The family history was significant for a paternal grandfather with BC and her older brother died of PC at 49 years of age. Mammography showed pleomorphic calcifications distributed segmentally with distortion on the right side (Fig. 1). Ultrasonography of the breast showed a 53 × 16 × 50 mm, ill-defined, low echogenic mass with calcifications in the right lower outer quadrant (Fig. 2). An ultrasonography-guided core needle biopsy demonstrated invasive ductal carcinoma. MRI of the breast showed multiple enhanced nodules on the right side (Fig. 3A). Swelling and enhanced axillary lymph nodes were also detected (Fig. 3B). PET/CT revealed FDG accumulation in the pancreas head as well as the right breast and axillary lymph nodes (Fig. 4). Therefore, MRCP was performed, which revealed a hypointense and hyperintense pancreatic mass on T1- and T2-weighted imaging, respectively (Fig. 5). The mass was 15 mm in size and dynamically enhanced on late phase (Fig. 5). A CT scan showed gradual attenuation and enhancement compared to the background pancreatic parenchyma (Fig. 6). There was no stenosis or dilation of the pancreatic duct (Fig. 6). EUS-FNA demonstrated PC. We provided genetic counseling and performed *BRCA* genetic testing, which revealed a heterozygous, deleterious mutation [NM_000059.4(*BRCA2*): c.9076C > T(p.Gln3026Ter)]. There were no abnormal findings on the gynecologic examination. She was diagnosed with premenopausal BC (cT3N2aM0, cStage IIIA, luminal type), HBOC, and PC (cT1cN0M0, cStage IA) and refereed to a university hospital.

At the university hospital, a combination chemotherapy regimen consisting of oxaliplatin, irinotecan, fluorouracil, and leucovorin (FOLFIRINOX) was first used to treat the PC. Next, a right breast mastectomy and axillary lymph node dissection were performed. The pathologic diagnosis of the permanent BC specimen was ypT2N1aM0, Stage IIB. The response to chemotherapy was grade 1. The histology was invasive ductal carcinoma, scirrhous type. The biological type was luminal type. One month after the mastectomy, a subtotal stomach-preserving pancreatoduodenectomy was performed. The pathologic stage for PC was ypT1bN0M0, ypStage IA. The response to chemotherapy was grade 2. The histology was invasive ductal carcinoma, adenocarcinoma, well-differentiated type. It was completely resected. She is alive without no recurrence.

## Discussion

We determined the association between the *BRCA2* variant in our patient and the onset of BC and PC. According to the ClinVar, the *BRCA2* variant has been reported for patients with PC, BC, and ovarian cancer [3]. The variant also has been reported in patients with PC alone. These findings suggest that the *BRCA2* variant detected in our patient was closely associated with the onset of PC.

We consider the surveillance utility of high-risk individuals with an inherited predisposition to PC, such as individuals with HBOC. According to the International Cancer of the Pancreas Screening Consortium, the aim of screening is to detect early, potentially curable PC [4]. In general, the rate of localized PC is 11% at the time of presentation and the rate of resectable PC is 15% [5]. The rate of resectable PC increases to 60–90% in patients who have been screened [5]. Furthermore, the 5-year survival rate for screening programs has improved to 60% compared to 10% in sporadic cases [5]. These findings suggest that surveillance of patients with a high PC predisposition is useful to improve prognosis. Because our patient had a high PC predisposition (first-degree relatives with PC and a PC-related genetic pathologic variant of *BRCA2*), active surveillance for PC was warranted.

The appropriate test for pancreatic surveillance should be considered. The National Comprehensive Cancer Network guidelines have recommended annual contrast-enhanced MRI/MRCP and/or EUS for patients at high risk for PC [2]. In contrast, the role of PET/CT in screening patients at high risk for PC has not been established. The Japan Study Group on the Early Detection of Pancreatic Cancer has reported that FDG accumulation of PET occurred in 1/11 (9.1%) stage 0 and 30/50 (60.0%) stage I patients [6]. The MRI findings were available in 46/51 (90.2%) stage 0 and 127/149 (85.2%) stage I patients [6]. EUS findings were available in 41/51 (80.4%) stage 0 and 132/149 (88.6%) stage I patients [6]. CT findings were available in 50/51 (98.0%) stage 0 and 146/149 (98.0%) stage I patients [6]. In our patient, PET/CT was performed for staging of BC. PC was incidentally detected by PET/CT and the lesion was confirmed by MRI and EUS. Because the superiority of PET-CT compared with MRI or EUS in detecting early-stage PC has not been demonstrated, MRI and/or EUS should be considered as first-choice imaging. Indirect findings, including main pancreatic duct dilation or stenosis, are often noted on CT and/or MRI. Furthermore, pancreatic uncus lesion often does not show these finding and it is difficult to detect PC. PET/CT could compensate for shortcomings of CT and/or MRI. Therefore, we should consider making good use of PET/CT, especially in individuals at high risk for PC, such as those with HBOC.

The cases of PC and BC detected simultaneously are summarized (Table 1) [7–13]. PC was first identified by CT or MRI in all cases and there were no cases detected by PET incidentally. Their stage of PC was more advanced than that of our case, and most cases showed not good prognosis. Therefore, our case could be a rare case in that incidental detection of PC by PET led to good prognosis.

**Table 1:** The cases of pancreatic cancer detected simultaneously with breast cancer

Authors, Year	Case	Detection method	Stage	Prognosis	Genetic test
Ofri A, et al [7], 2022	61 yo female	PC:MRI⇨PET, EUS BC:PET	PC: pStage IIA BC: pStage IIIC	She underwent upfront surgery for PC and BC. PC recured 5 month post operatively	*BRCA1/2*: negative *PALB2*: negative
Ofri A, et al [7], 2022	77 yo female	PC: CT BC: CT	PC: unknown BC: 2 left breast lesions (19 mm and 8 mm in size)	She underwent NAC for PC and neoadjuvant endocrine therapy for BC. Liver metastases developed just before surgery	negative
Nakanishi K, et al [8], 2021	78 yo female	PC: CT⇨EUS BC: US, CT	PC: cStage IIA BC: cStage IIB	NAC → Surgery for PC and BC → Adjuvant chemotherapy no recurrence	not assessed
Takada K, et al [9], 2018	67 yo male	PC: CT, MRI BC: CT, US	PC: cStage IV BC: cStage IIA	Lever failure developed and best supportive care was begun	not assessed
Castro M, et al [10], 2016	41 yo female	PC: CT, PET BC: MMG, US, MRI	PC: pStage IIB BC: pStage IIIA	Mastectomy → Surgery for PC → adjuvant chemotherapy, radiation therapy for BC and PC, and endocrine therapy. Alive at the time of publication	germline deleterious *BRCA2* variant
Kim JS, et al [11], 2013	73 yo female	PC: CT, EUS, MRI, PET BC: PET, US	PC: 1.5 × 1.8 cm mass with celiac axis invasion and the main pancreatic duct dilation BC: several masses in the left side	Treated by hormone therapy for BC and provide supportive care for PC. Died 8 months after the diagnosis	not assessed
Unek IT, et al [12], 2008	50 yo male	PC: MRI, PET BC: MMG, US, PET	PC: 25 mm mass BC: 13 mm mass in the right side	Upfront surgery for PC and BC. Post-operative radiotherapy to chest wall as well as the pancreatic region. Chemotherapy was also done. After 6 cycles of chemotherapy, metastases to lung and liver developed. Died 10 months after the diagnosis	not assessed
Morganti AG, et al [13], 2008	69 yo male	PC: CT BC: US	PC: 30 mm in size with liver metastasis BC:25 mm and 15 mm masses in right side	Mastectomy followed by chemotherapy and hormone therapy were done. Died 17 months after the diagnosis of PC and BC	not assessed

*PC* pancreatic cancer, *BC* breast cancer, *yo* year-old, *NAC* neoadjuvant chemotherapy, *MRI* magnetic resonance imaging, *CT* computed tomography, *EUS* endoscopic ultrasonography, *PET* positron-emission tomography, US ultrasonography, *MMG* mammography

Cost-effectiveness of imaging-based PC screening methods is an important issue. Kowada reported it in familial high-risk individuals (HRIs) of PC in Japan [14]. Abdominal ultrasound was the most cost-effective and recommended for PC screening in familial HRIs in Japan [14]. It contributed to increasing quality-adjusted life-years, decreasing cancer mortality and reducing the costs by detecting early PC [14]. Since cost-effectiveness was influenced by the incidence of PC, EUS and MRI became more cost-effective than abdominal ultrasound when the incidence of PC was high [14]. The report has also proposed that the physician should perform MRI or EUS, if it was difficult to find PC by abdominal ultrasound [14]. Meanwhile, PET and CT were high incremental cost-effectiveness ratio, less effective and more costly than others [14]. Therefore, PET/CT should not be performed constantly in order to detect PC even if a patient is HBOC.

In conclusion, investigation of PC should be considered for patients with HBOC. Although MRI and/or EUS should be considered as first-choice imaging, PET/CT could be a potent tool for surveillance.

## Data Availability

The data that support the findings of this study are not openly available to protect study participant privacy and are available from the corresponding author upon reasonable request. Data are located at Matsue City Hospital.

## Abbreviations

PET/CT: F-18-fluorodeoxyglucose positron emission tomography / computed tomography ()
HBOC: Hereditary breast and ovarian cancer syndrome
PC: Pancreatic cancer
BC: Breast cancer

## Declarations

### Human right

It is released in according with the ethical standards as laid down in the 1964 Declaration of Helsinki and its later amendments or comparable ethical standards.
